# Properties of Concrete with Recycled Concrete Aggregate Containing Metallurgical Sludge Waste

**DOI:** 10.3390/ma13061448

**Published:** 2020-03-22

**Authors:** Jan Pizoń, Jacek Gołaszewski, Mohamed Alwaeli, Patryk Szwan

**Affiliations:** 1 Faculty of Civil Engineering, Silesian University of Technology, 44-100 Gliwice, Poland; jacek.golaszewski@polsl.pl (J.G.); szwan.patryk@gmail.com (P.S.); 2 Faculty of Energy and Environmental Engineering, Silesian University of Technology, 44-100 Gliwice, Poland; mohamed.alwaeli@polsl.pl

**Keywords:** concrete, aggregate, recycled concrete aggregate, recycled aggregate concrete, concrete properties, metallurgical sludge waste, circular economy

## Abstract

Sand has been considered to be something of an immeasurable quantity. There are many indications that this view is no longer valid and that the limiting of natural aggregates usage is doubly justified. Firstly, the extraction of natural aggregates is expensive and has a huge impact on the environment. The main issues in sand and gravel mining are the large areas that are affected, ground water level changes, illegal mining, unsuitability of desert and marine sand, and costs of transport. Secondly, metallurgical waste can be used as a substitute for natural aggregates. This is doubly beneficial—the waste is recycled and the use of natural aggregates is reduced. Waste is stored in landfills that take up large areas and there is also the possibility of ground and groundwater pollution by hazardous compounds. The research presented in this article focuses on the technological conditions of using metallurgical waste in its original form and as a component of recycled concrete aggregate (RCA). The use of metallurgical sludge waste or crushed or round RCA to produce concrete deteriorates the consistency and does not significantly affect the air content and density of the concrete mix. RCA lowers the density of hardened concrete. Metallurgical sludge waste or RCA usage adversely affect the absorbability and permeability of concrete. Concrete containing metallurgical sludge waste is of higher compressive strength after 7 and 28 days, with up to 60% of waste as a sand replacement. RCA concrete achieved higher compressive strength also.

## 1. Introduction

Global production of aggregates was 21 B tonnes in 2007 and 40 B tonnes in 2014 [1,2]. The most recent data presents value of 50 B tonnes of sand and gravel yearly consumption [3]. According to Big Market Research (BMR), Aggregates Market Development by 2026 report [4] and Grand View Research (GVR), Aggregates Market Size, Share and Trends Analysis Report [1] the global aggregates market was worth about 430 B USD in 2018, and is still raising. It is predicted to be worth about 600 B USD in 2026. The GVR’s report showed that about 60% of aggregates excavated worldwide was used for concrete production and about 20% for road substructures [1]. It was estimated that only about 100 M tonnes of concrete were recycled into aggregate in 2004 [5]. In 2017 only in Great Britain 72 M tonnes of recycled and secondary sources aggregates were used, which is quite a large amount in comparison to 176 M tonnes of primary aggregates [6]. Nowadays, limiting the use of natural aggregates to concrete is doubly justified.

Firstly, natural aggregates are, as the name suggests, a natural resource. Their extraction is expensive and has a huge impact on the environment [2,7,8]. Sand and gravel mines occupy very large areas and cause a change in the groundwater level in their vicinity [8]. Additionally, sand mining in some countries is subject to prohibitions or very high taxes [9,10]. Black market and illegal sand extraction exist for example in Italy, India, Vietnam, Malaysia and others [9,11,12]. In other desert countries (e.g., Egypt, Algeria, United Arab Emirates), although there is a lot of sand, it is not suitable for use in concrete, due to the high fineness and too smooth texture of the grains [2,9,12,13] or the pollution of sea sand chlorides [11]. However, both fields are studied [13,14,15,16].

Secondly, metallurgical waste—sludge, slag and dust from blast-furnace, steel and sintering processes can be used as a substitute for natural aggregates. This is doubly beneficial—the waste is recycled and the use of natural aggregates is further reduced. This waste is deposited in landfills that take up large areas. There is also a danger of hazardous compounds leaking to the ground and groundwater [17,18,19,20].

Therefore, it is worth considering the recovery and use of other materials as an alternative to natural aggregates—various types of industrial waste and demolition rubble from buildings such as bricks or concrete [21,22]. 

Recycled concrete aggregate (RCA) is a shredded waste generated during the demolition of concrete and reinforced concrete structures. The resulting aggregates are the subject of research to reduce the need for natural aggregates for concrete applications.

The use of RCA increases the demand for water and decreases the consistency of the concrete mix with the same effective water-cement ratio. This applies to mixtures containing 50% and 100% RCA [23]. Similar results are presented in [24]. The studies described in [25] have shown that mixes containing 50% and 100% RCA and modified with a superplasticizer have reached the same consistency as the reference mix-therefore it is possible to control the consistency of mixes containing even large amounts of RCA.

No effect of the substitution of 50% and 100% RCA on the air content of the concrete mix was found [23]. The studies described in [25] showed that mixes containing 50% and 100% RCA show a slightly increased air content.

The full replacement of the natural aggregate with RCA results in a lower concrete mix density [23]. The density of High Performance Concrete (HPC) containing RCA is lower compared to the control mix [26]. The tests described in [25] showed that mixtures containing 50% and 100% RCA have lower density in comparison with the control mixture.

The use of RCA may result in deterioration of the mechanical properties of concrete—this depends on the quality of the concrete used. It is stated that the use of 30% RCA as a substitute for natural aggregate has not resulted in a decrease in compressive strength [27]. Increasing the amount of RCA to 100% reduced strength by about 10%. The strength of concrete containing 100% RCA is lower by about 8% after 2 days but higher by about 5% after 7 and 28 days [23]. After 28 days a decrease in compressive strength by about 10% was found [24]. Similar relationships were found for mortars containing 25%–100% fine RCA [28]. Reduction of compressive and flexural strength and elasticity modulus was also described for HPC containing RCA after 7, 14 and 28 days [26]. Concretes containing 50% and 100% RCA have lower compressive strength compared to control concrete, however, the use of concrete from railway sleepers demolition caused an increase in strength [25]. The compressive strength of concrete containing RCA is 30% less than the reference concrete, and the compressive strength is 10% less. Static and dynamic modulus of elasticity decreased by about 20% [29]. Similar results were obtained by other researchers [30].

The use of RCA as a replacement for natural aggregate results in increased concrete absorbability. The absorbability increases from about 5.5% for concrete without RCA to about 8% at 100% replacement level. Increase of absorbability and porosity by 15%–38% and decrease of density by 4%–7% for HPC is described in [26]. Studies described in [25] showed that concretes containing 50 and 100% RCA have a lower density compared to control concrete. The density of concrete containing 100% RCA is lower than the reference concrete by 5%–6% and its absorbability higher by about 70% [29].

The capillary action and permeability of concrete containing RCA is increased by about 20% compared to the reference concrete [24]. The permeability of concrete containing RCA can be reduced to a lower level than for concrete with natural aggregates using supplementary cementitious materials such as phosphorus slag, blast-furnace slag [31].

The use of fine RCA causes a decrease of the durability properties of concrete—an increase of absorbability and permeability, increase of carbonation depth and chloride migration rate [32]. However, it is possible to shape and improve these properties using chemical admixtures—mainly superplasticizers. No significant decrease in durability properties of concrete using up to 30% RCA was found [33]. Above this amount a faster corrosion of reinforcing steel was observed. Chloride diffusion coefficient increased in concretes containing RCA [30,34].

Other uses of the RCA include: roads substructures, earth dam construction [35,36,37], paving blocks and structural blocks and bricks manufacture [37,38], bituminous-aggregate mixes [39,40,41,42], road pavements [43], self-compacting concrete [44,45,46], polymer concrete [47], shotcrete [48,49], concrete exposed to elevated temperatures [50], soil stabilization [51], pervious concrete [52], reactive powder concrete [53], artificial reef formation [37].

Recycled aggregate concrete (RAC) is a concrete in which all or part of the natural aggregate is replaced by waste materials [54]. Many researchers have drawn attention to the problem of limiting the use of natural aggregates and have tried to use different materials as a substitute. 

A lot of waste from the metallurgical industry has been involved in concrete technology. The main example is the use of ground granulated blast furnace slag and fly ash as a component of cement and concrete [55]. Blast furnace slag, electric arc furnace slag, fayalite slag, basic oxygen furnace slag, and copper slag are widely used as an aggregate for concrete [56,57,58,59,60,61,62,63,64,65,66,67]. Other metallurgical wastes such as steel scales, chips and iron ore wastes are used as well [68,69,70].

Another steel making process by-product is sludge. The use of sludge as an aggregate or aggregate component is not widely recognized. It is related to the fineness of the material causing a huge increase in water demand and the content of heavy metals, which may cause an extension of the initial setting time of the cement [71]. 

Thermally treated steelmaking sludge was used to manufacture the lightweight aggregate [72]. Sludge was sintered with clay to create pellets—results are promising but pellets manufacture is a high energy consuming process.

The research presented in this article focuses on the technological conditions of using metallurgical waste in its original form and as a component of RCA. Such an approach to the possibilities of metallurgical waste management has not been widely studied before.

Metallurgical sludge waste was added to concrete in three forms:

- Dried and ground material (see Section 2.1)
- Component of rounded RCA as a partial replacement of fine aggregate (see Section 2.2)
- Component of crushed RCA as a partial replacement of fine aggregate (see Section 2.3)

The former method is generally suitable for manufacturing of non-structural concrete elements, weak concrete layers or curb fixing, etc. Both latter methods may be used in situation while the concrete mix is left on the building site or the concrete do not meet requirements and need to be recycled. Then the metallurgical sludge waste together with admixture is added to concrete mix to form RCA. Additionally, the second method may be used to recycle elements manufactured with concrete containing metallurgical sludge waste and reuse it as RCA after crushing.

## 2. Materials and Methods

### 2.1. General Information

During research concrete with RCA containing metallurgical sludge waste, concrete with metallurgical sludge waste as a partial replacement of fine aggregate and the reference concrete were prepared. RCA was manufactured in two forms—rounded and crushed.

For the preparation of RCA containing metallurgical sludge waste and all concrete types following materials were used: Portland cement, natural coarse and fine aggregate, metallurgical sludge waste, admixtures (superplasticizer and concrete mix recovery agent) and tap water. All ingredients were stored in temperature 20 ± 1 °C.

Portland cement CEM I 42.5 R that is complying with requirements of EN 197-1:2012 was used. Phase composition of cement is given in Table 1.

**Figure 1:**
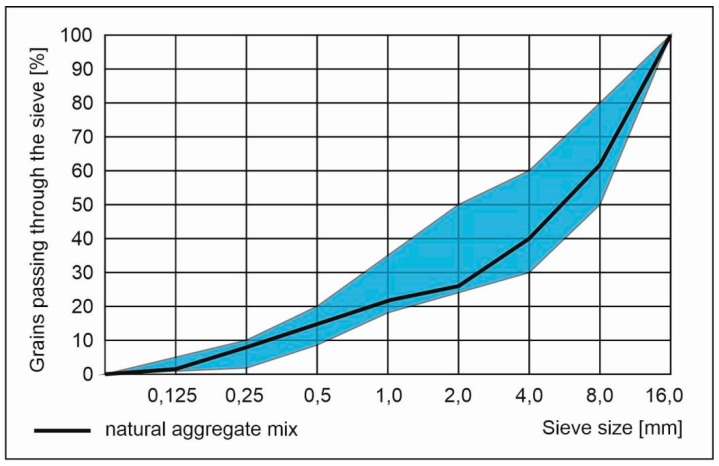
Grading of natural aggregate mix. Blue regions within the limiting curves denotes the most suitable aggregate’s grading for concrete.

Natural aggregate both coarse and fine were used. Coarse aggregate that was used is natural gravel and fine one was a natural quartz sand. Aggregate mix was composed of three separate fractions 0–2, 2–8 and 8–16 mm. For the grading curve of the aggregate mix see Figure 1. Admixtures involved in research were:

- Superplasticizer containing polycarboxylate ethers used for consistency enhancement. It was used both for crushed and rounded RCA preparation and execution of concrete with dried metallurgical sludge waste and containing RCA.
- Concrete mix recovery agent—two-component, powdered special agent for recovery of concrete mix and form rounded RCA.

### 2.2. Metallurgical Sludge Waste

**Figure 2:**
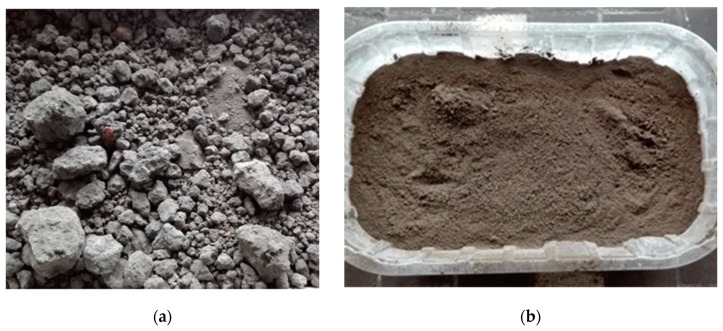
Metallurgical sludge waste used in research (**a**) unprocessed, (**b**) oven dried, ground.

Metallurgical sludge waste used in research was obtained from one of polish steel manufacturers. As by-product it was stored on the landfill. It consists of sintering sludge, blast furnace sludge and converter sludge in varying proportions. The appearance of unprocessed and ground dried metallurgical sludge waste is shown in Figure 2. Processed metallurgical sludge waste is a very fine powdered material that consists of grains 0–0.25 mm. Comparison of physical properties of natural sand and metallurgical sludge waste is given in Table 2.

Chemical composition of metallurgical sludge waste from this landfill depends on exact point from which it was taken. Chemical composition is given in Table 3. Metallurgical sludge waste was tested for the reactivity by micro calorimetry method. There was no evidence that heat is exhaled during its reaction with water. Thus, we use this material as an inert filler. All metallurgical wastes in form of slag, scale, or sludge are considered to be hazardous materials. One of possibilities of reuse of such waste is incorporating them into the concrete as a replacement of natural aggregate. The main issue with utilization of metallurgical sludge waste is its zinc content. It leads to retardation of cement hydration reaction. It reacts with tricalcium silicate [73] and produces Ca(Zn(OH)_3_)_2_∙2H_2_O phase [74].

### 2.3. Preparation of Recycled Concrete Aggregate

**Figure 3:**
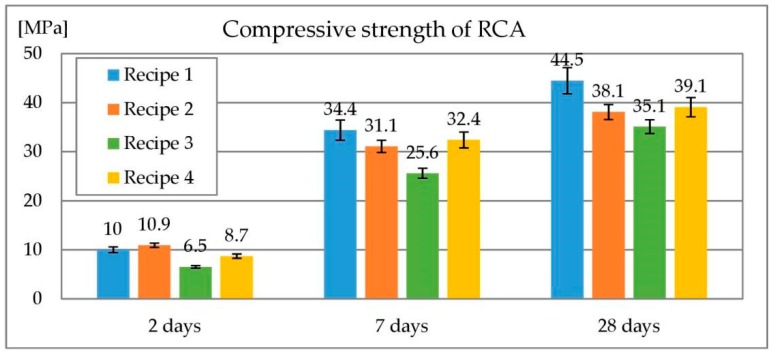
Compressive strength of preliminary recipes for RCA.

Both rounded and crushed RCA were prepared using the same recipe. The only difference is presence of concrete mix recovery agent in former one. Four preliminary recipes were prepared to obtain optimal proportions of components. Description of mixes used for RCA preparation are given in Table 4. Recipe 1 was considered best according to its compressive strength after 28 days. Results of compressive strength of preliminary RCA recipes are given in Figure 3.

**Figure 4:**
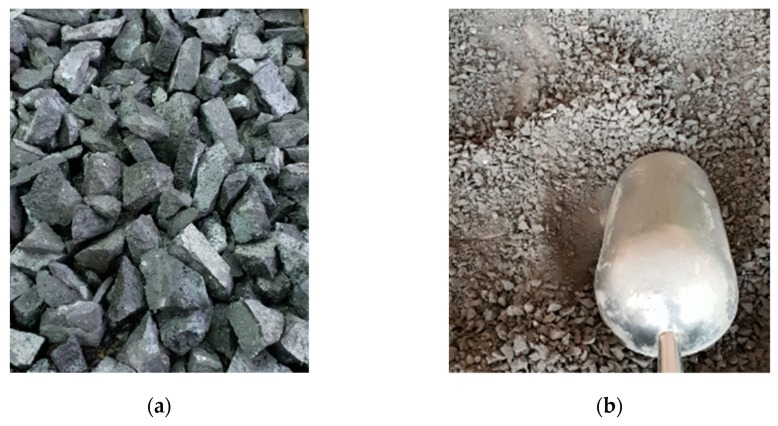
Crushed recycled concrete aggregate: (**a**) hand crushed, (**b**) mechanically crushed.

**Figure 5:**
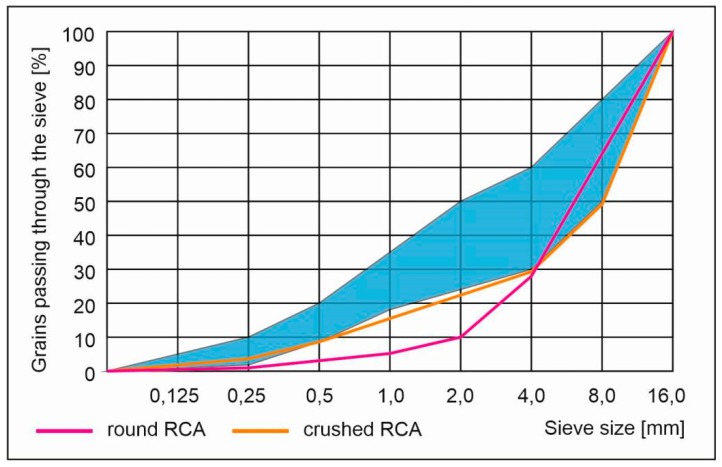
Grading of recycled concrete aggregate. Blue regions within the limiting curves denotes the most suitable aggregate’s grading for concrete.

Stages of crushed RCA manufacture:

- Preparation of mix according to recipe 1 (Table 4) and formation into prisms.
- Cover with foil for first 24 h to avoid excessive evaporation of water.
- Curing of composite for next 27 days in water at temperature 20 ± 1°C.
- Hammer crushing of aggregate to grain size <60 mm (see Figure 4a).
- Mechanical crushing of aggregate (see Figure 4b).
- Sieve analysis of crushed RCA (see Figure 5).

**Figure 6:**
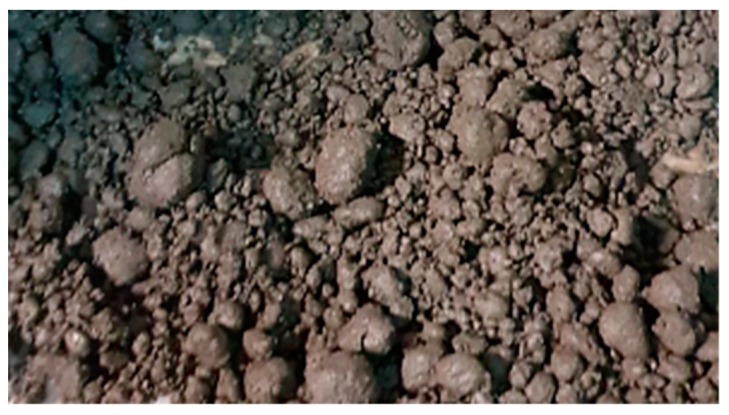
Crushed recycled concrete aggregate.

Stages of rounded RCA manufacture:

- Preparation of mix according to recipe 1 (Table 4) in free fall concrete mixer.
- Mixing for 3 min at high speed after adding concrete mix recovery agent component A (0.5 kg for 1 m^3^ of concrete mix).
- Mixing for 2 min at medium speed after adding concrete mix recovery agent component B (6 kg for 1 m^3^ of concrete mix).
- Spreading of granules in one layer on the flat surface (see Figure 6). Granules were cover with foil for first 24 h to avoid excessive evaporation of water. During this time material was manually stirred to avoid gluing particles together. Procedure of stirring was repeated 4 times in 6 h interval.
- Curing of composite for next 27 days in water at temperature 20 ± 1°C.
- Sieve analysis of crushed RCA (see Figure 5).

Both natural aggregate and RCA were tested for density and absorbability. Results are given in Table 5.

### 2.4. Concrete Mix Preparation

Three series of concrete were produced during the research. Compositions of concrete mixes are given in Table 6. For the preparation of first series (symbol MSW XX, where XX denotes replacement level), dried, ground metallurgical sludge waste was used as a partial replacement of sand. Replacement levels were established as 30%, 60% and 90%. The second and third series involved recycled concrete aggregate containing metallurgical sludge waste. One was produced with crushed RCA (symbol CR XX, where XX denotes replacement level) and the other with rounded one (symbol RO XX, where XX denotes replacement level). RCA were used as a partial replacement of the natural coarse aggregate. Replacement levels were 25%, 50% and 75%. Reference concrete was produced as well.

### 2.5. Methods

The following tests were conducted for concrete mixes: air content, consistency and density. Hardened concrete samples in form of cubes 15 × 15 × 15 cm^3^ was tested for compressive strength, density, permeability and water absorption. Samples were cured in water at 20 ± 1 °C. Compressive strength tests were carried out after 2, 7 and 28 days. Water absorption, permeability and density were tested after 28 days.

Tests were conducted according to standards:

- Sieve analysis of aggregates: PN-EN 12620+A1:2010,
- Consistency of concrete mix: PN-EN 12350-2:2011,
- Air content in concrete mix: PN-EN 12350-7:2011,
- Density of concrete mix: PN-EN 12350-6:2011,
- Compressive strength of concrete: PN-EN 12390-3:2011,
- Water absorption of concrete: PN-EN 13369:2018-05,
- Density of concrete: PN-EN 12390-7:2011,
- Permeability of concrete: PN-EN 12390-8:2011.

## 3. Results and Discussion

Research has been carried out on concretes containing metallurgical sludge waste in the least processed form—dried and ground (Section 3.1) and enclosed in concrete and used as RCA (Section 3.2). The research was aimed at testing the technological possibilities of using metallurgical sludge waste in various forms as a partial replacement for aggregate parts in concrete.

### 3.1. Concrete with Metallurgical Sludge Waste as A Partial Sand Replacement

#### 3.1.1. Consistency

**Figure 7:**
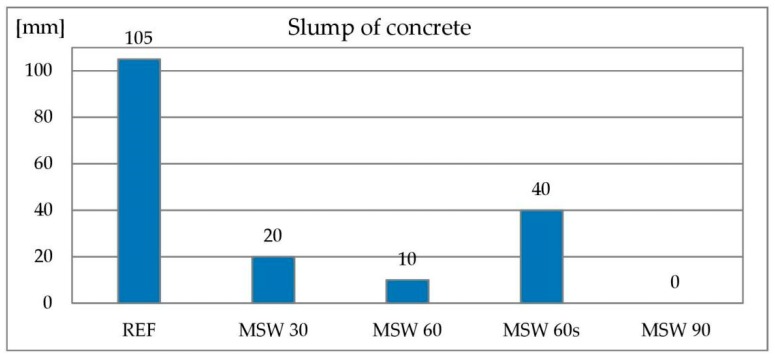
Slump of concrete with metallurgical sludge waste.

The consistency tests were conducted to determine the effect of the metallurgical sludge waste content on the plasticity of the concrete mix. It was suspected that due to the fineness of the material and its high water demand the consistency would deteriorate. The reference mixture showed a slump of 105 mm, which qualifies it to consistency class S3 according to EN 206-1. Results of consistency tests are presented in Figure 7. Concrete containing metallurgical sludge waste as 30% sand replacement reached 20 mm slump and the one containing 60% of metallurgical sludge waste as a sand replacement 10 mm. An attempt was made to improve the consistency of the latter with a superplasticizer. After the addition of a 1.55% admixture by cement’s mass, the slump reached 40 mm. As a result, these three mixes reached the S1 consistency class. The concrete mix containing metallurgical sludge waste as a 90% sand replacement did not show a slump even after adding a superplasticizer in amount of 2% of cement mass, which is the maximum dosage recommended by the manufacturer. It cannot be classified in any of the consistency classes. Deterioration of consistency is related to the high water demand of metallurgical sludge waste and its high granularity. However, with the help of superplasticizers it is possible to freely shape the consistency of a mixture containing large amounts of metallurgical sludge waste in such a way that it can be applied to the civil engineering purposes. The effect of consistency loss of the concrete mix containing metallurgical sludge waste can be compared to the use of other dusts and powders. One example is the granite dusts described in [75], which greatly increase the amount of water required to make the concrete mix workable. Similar observations are described for concretes containing glass powder [76], marble powder [77,78], quartz powder, dolomitic powder and other stone dusts [79] and sewage sludge waste [80]. Studies described by other researchers confirm that it is possible to shape the consistency of a dust-containing mixes freely with the superplasticizers [75].

#### 3.1.2. Air Content

**Figure 8:**
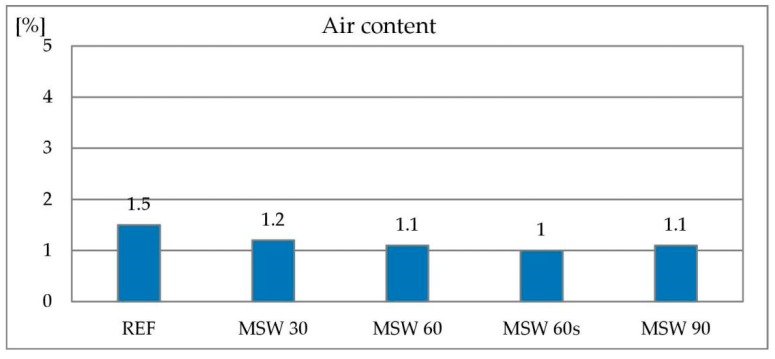
Air content in concrete mix with metallurgical sludge waste.

The measurement of air content in the concrete mix was to determine whether the concrete mixes, despite their deteriorated consistency, are capable of adequate compaction and deaeration after application of vibrations. The reference mix had 1.5% air content. All mixes containing metallurgical sludge waste showed a lower air content (1.0%–1.5%) in comparison to the reference mix. However, this is not a significant difference and can be related to the vibration time and accuracy of the test method. The results of the air content in concrete mixes tests are shown in Figure 8. It was proved that it is possible to properly compact and deaerate mixes containing metallurgical sludge waste, of course after applying a superplasticizer to improve consistency.

#### 3.1.3. Density of Concrete Mixes

**Figure 9:**
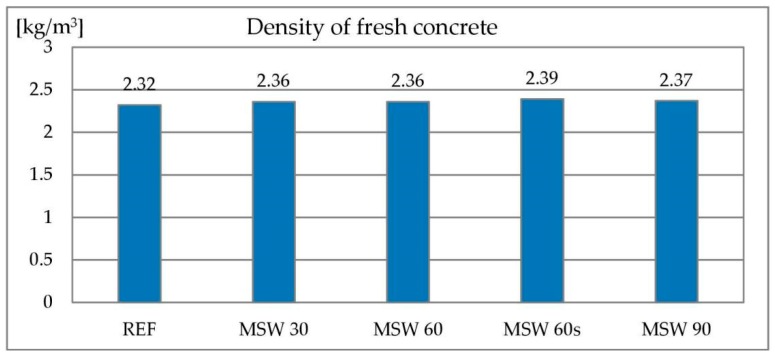
Density of concrete mix with metallurgical sludge waste.

The density of the concrete mixture is related to its air content. The reference mixture had a density of 2.32 kg/dm^3^. All mixes containing metallurgical sludge waste showed a slightly higher density (2.36–2.39 kg/dm^3^). This is caused by a higher degree of structure tightness by introducing a very fine material. The degree of compactness can be significantly higher due to the large difference in density between metallurgical sludge waste and sand, which is significantly lower for the former material. The results of the concrete mix density test are shown in Figure 9.

#### 3.1.4. Density of Hardened Concrete

**Figure 10:**
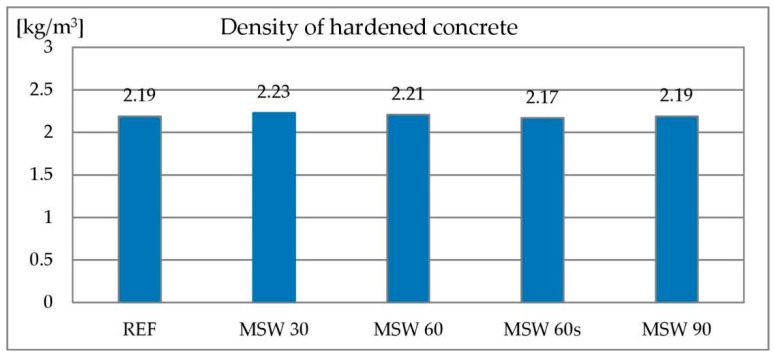
Density of hardened concrete with metallurgical sludge waste.

Density of hardened concrete is presented in Figure 10. No significant differences were found between the reference concrete and those containing metallurgical sludge waste. All concrete samples had a density of approximately 2.2 kg/dm^3^. This similarity is connected to denser structure of concrete containing metallurgical sludge waste, despite of lower density of metallurgical sludge waste itself in comparison to sand that it replaced.

#### 3.1.5. Water Absorbability

**Figure 11:**
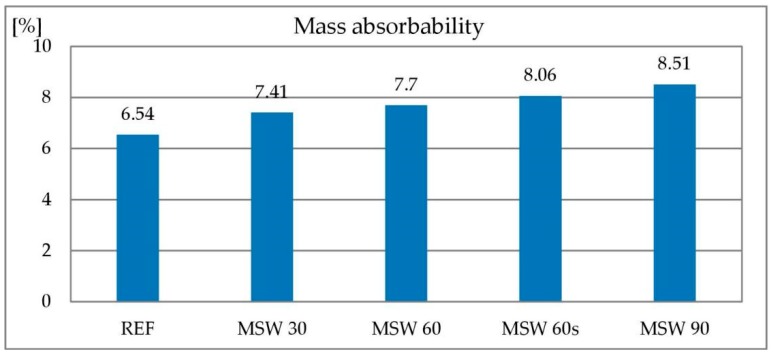
Absorbability of concrete with metallurgical sludge waste.

Concrete is a porous material. The pores it contains, in particular the capillary ones, are filled with water in damp and wet environments. Concrete’s absorbability is one of the properties affecting its durability—the higher the absorbability, the greater the possibility of penetration of aggressive chemicals dissolved in water. Testing the absorbability of concrete consists in determining the amount of water that concrete is able to absorb until the pores are completely filled. The absorbability of the reference concrete was 6.5%. With an increase in the content of metallurgical sludge waste, the absorbability of the concrete increased up to 8.5% when 90% of the sand was replaced by metallurgical sludge waste. The air content study showed no difference between the individual concrete mixes that could explain this relationship. This means that during the hardening and drying of the concrete, water evaporates not only from the cement matrix but also from the metallurgical sludge waste, which is part of the aggregate. This can be caused by the absorbability of the aggregate, which is considerably higher for metallurgical sludge waste compared to sand. The results of the concrete absorbability test are shown in Figure 11.

#### 3.1.6. Permeability

**Figure 12:**
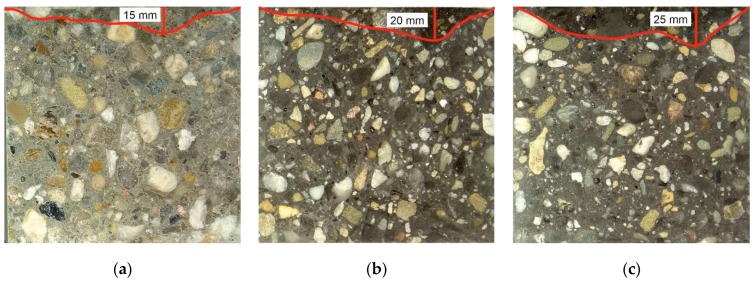
Permeability of (**a**) reference concrete, (**b**) concrete with 30% of metallurgical sludge waste, (**c**) concrete with 90% of metallurgical sludge waste.

The permeability of concrete is related to its density and absorbability. It is defined as the depth of water penetration under pressure. Apart from permeability, it is another factor influencing the durability of cement composites. Figure 12 shows the penetration depth of water in samples: (a) reference and (b) containing 30% metallurgical sludge waste as a replacement for sand and (c) containing 90% metallurgical sludge waste as a replacement for sand. The penetration depth of water increases with the amount of metallurgical sludge waste. At 90% of metallurgical sludge waste it is 25 mm. This is not a major difference compared to the reference sample for which penetration depth is 15 mm. It can therefore be assumed that the content of metallurgical sludge waste can affect the durability of the concrete, but this will not have a very strong impact.

#### 3.1.7. Compressive Strength

**Figure 13:**
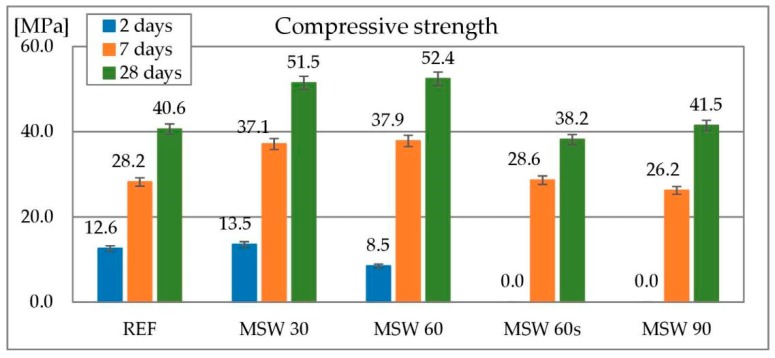
Compressive strength of concrete with metallurgical sludge waste.

Compressive strength of concrete is one of the most important properties of concrete. The results of compressive strength tests of concretes containing metallurgical sludge waste as a replacement of sand is shown in Figure 13. The compressive strength of concretes containing metallurgical sludge waste after 2 days of curing was highly dependent on metallurgical sludge waste amount. In the case of the concrete with 30% sand replacement compressive strength was similar to the reference sample. For 60% substitution without superplasticizer (MSW 60) it was significantly lower (for about 40%). Finally, in the case of the both concretes containing superplasticizer (MSW 60s and MSW 90), it was not possible to examine the compressive strength after two days as the samples did not show sufficient strength to be demolded. After a longer curing time (7 and 28 days), concrete in which 30 and 60% of fine natural aggregate was replaced by metallurgical sludge waste showed much higher compressive strength than the reference concrete. For MSW 30 and MSW 60, the strength was about 30% higher than the reference concrete. Both remaining concretes (MSW 60s and MSW 90) had very similar compressive strength results to the reference concrete. Such behavior, which is manifested by an increase in the strength of the concrete despite the use of the matallurgical sludge waste, as a substitute for sand, is connected with the efficient water-cement ratio which is lowered by metallurgical sludge waste due to its high water demand and fineness. Similar results with other very fine materials were obtained with [77,78] for marble powder, [75] for granite powder and [76] for steel and glass powder.

### 3.2. Concrete with RCA Containing Metallurgical Sludge Waste as A Partial Replacement of Coarse Aggregate

#### 3.2.1. Consistency

**Figure 14:**
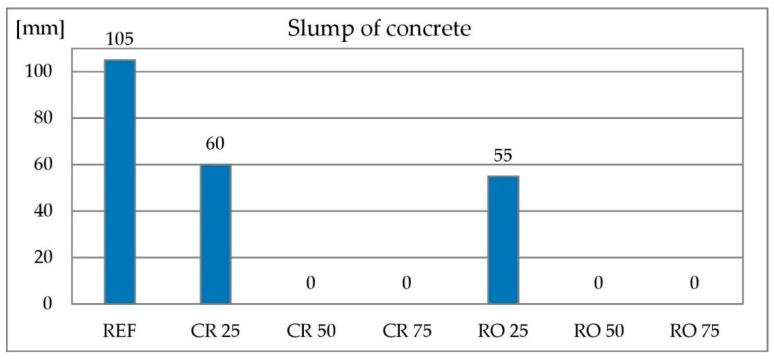
Slump of concrete with recycled concrete aggregate.

The reference mixture showed a slump of 105 mm, and is qualified aa S3 consistency class according to EN 206-1. Results of consistency tests are presented in Figure 14. Concretes with both crushed (symbol CR XX, where XX denotes replacement level) and round (symbol RO XX, where XX denotes replacement level) RCA as a 25% replacement of coarse aggregate show a 60 and 55 mm slump respectively. It classifies them into S2 consistency class. All concretes with higher replacement levels shown no slump at all, even though consistency of both concretes with 75% of coarse aggregate replacement was improved by superplasticizer. It is connected to higher water absorbability of RCA in comparison to natural gravel. Similar results are reported by [81] for crushed RCA created without addition of any waste—water demand of RCA is significantly higher than for natural aggregates. Deterioration of consistency in lower range for both crushed ceramic waste and RCA is reported by [82,83,84].

#### 3.2.2. Air Content

**Figure 15:**
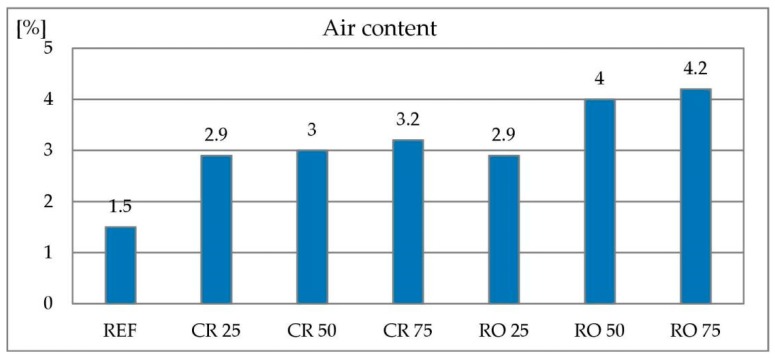
Air content of concrete with recycled concrete aggregate.

In concretes using crushed and round RCA as a replacement for natural aggregate, the amount of air in the concrete mix increases. Test results are given in Figure 15. However, in case of concrete with crushed RCA, there is no clear correlation between the air content and the amount of natural aggregate replaced. The increase in air content is due to the higher porosity of RCA compared to natural aggregate. In the case of concrete with a round RCA, the air content increases with the amount of natural aggregate replaced. In this case, the stiff consistency can also have an impact, making the mix more difficult to place in the porosimeter and more difficult to compact.

#### 3.2.3. Density of Concrete Mixes

**Figure 16:**
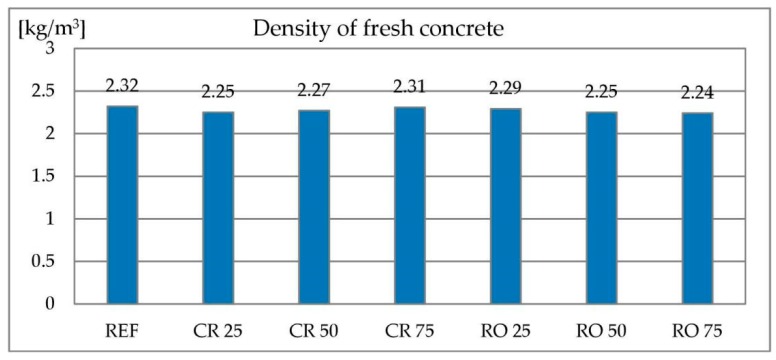
Density of concrete mix with recycled concrete aggregate.

For concrete mixes containing metallurgical sludge waste as a sand replacement, the density of concrete mix was independent of the replacement level. In case of concrete mixes containing RCA, the situation is similar. All mixes densities are in range of 2.24–2.32 kg/dm^3^. Densities of concrete mixes containing RCAs are given in Figure 16.

#### 3.2.4. Density of Hardened Concrete

**Figure 17:**
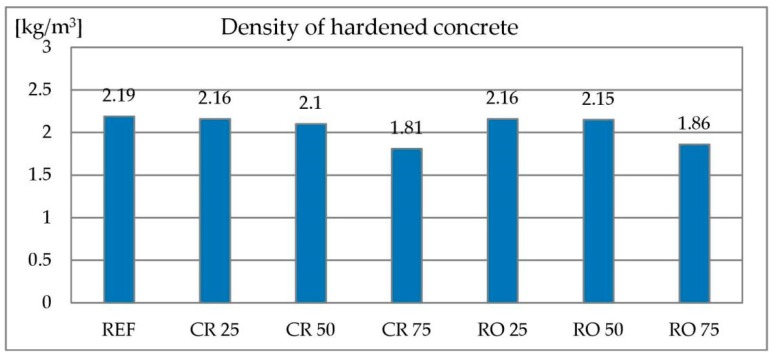
Density of concrete with recycled concrete aggregate.

In the case of concretes where sand was replaced by metallurgical sludge waste, the density of concrete was independent of the degree of replacement. In case of concretes containing RCA, the density decreases with increasing RCA content. It is shown in Figure 17. This is particularly evident when 75% of the sand was replaced by crushed or round RCA. RCA has a much lower density (2.2 kg/dm^3^) than natural gravel aggregate (2.65 kg/dm^3^)—this reduces the density of the composite. However, it is necessary to be aware of a much greater replacement level in terms of volume—here the coarse aggregate is changed and in the previously described case only sand, which occupies much less volume in the concrete mix.

#### 3.2.5. Water Absorbability

**Figure 18:**
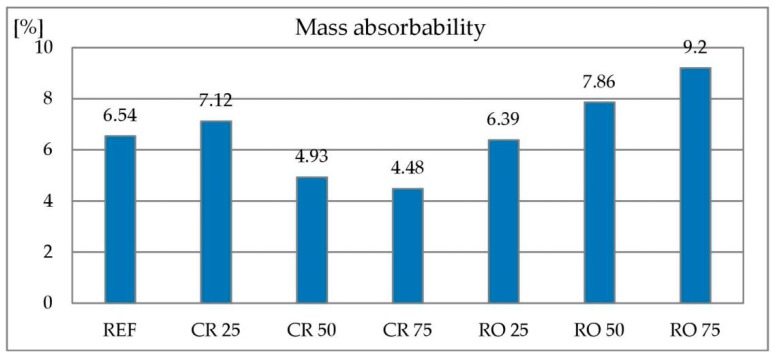
Absorbability of concrete with recycled concrete aggregate.

Water absorbability of concrete containing crushed RCA as 25% replacement of coarse aggregate is higher in comparison to reference concrete. However, with a greater replacement level, the absorbability decreases. This is surprising because of the higher absorbability of the RCA compared to natural aggregates. In the case of round RCA, the absorbability of concrete increases as the replacement of coarse aggregate by metallurgical sludge waste increases. Absorbability of concrete with coarse aggregate substitution by RCA is presented in Figure 18. Similar results are reported by [81,82,85] for crushed RCA created without addition of any waste.

#### 3.2.6. Permeability

**Figure 19:**
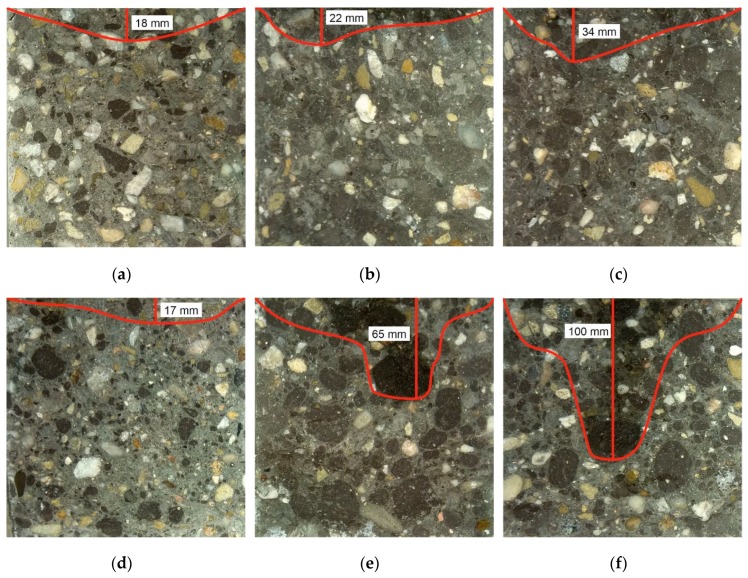
Permeability of (**a**) concrete with 25% crushed RCA, (**b**) concrete with 50% crushed RCA, (**c**) concrete with 75% crushed RCA, (**d**) concrete with 25% round RCA, (**e**) concrete with 50% round RCA, (**f**) concrete with 75% round RCA.

Permeability of concrete containing 25% and 50% crushed RCA replacement of gravel is similar to reference concrete. 75% of crushed RCA in concrete leads to increase of permeability. However, the increase is not very evident. Permeability of crushed RCA concrete is shown in Figure 19a–c. Permeability of concrete containing 25% rounded RCA replacement of coarse aggregate is similar to reference sample as well. Increase of rounded RCA content to 50 and 75% leads to evident increase of permeability. It is clearly visible that way of water going through the concrete is mainly through the RCA. It means that durability of such concrete may be significantly affected. Permeability of crushed RCA concrete is shown in Figure 19d–f.

#### 3.2.7. Compressive Strength

**Figure 20:**
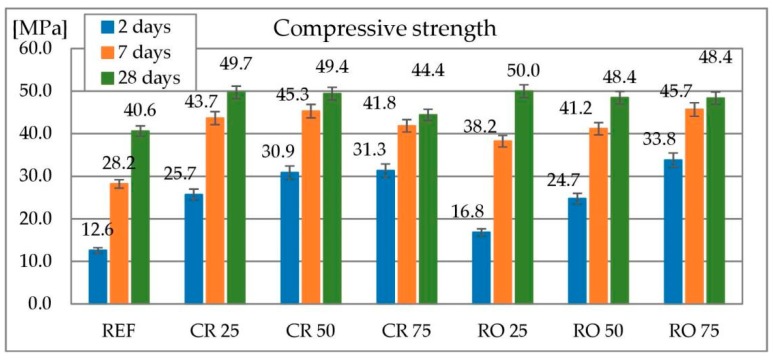
Compressive strength of concrete with recycled concrete aggregate.

The results of compressive strength tests of concretes containing both crushed and round RCA as a replacement of coarse aggregate is shown in Figure 20. At each testing date, concretes containing RCA as a replacement for coarse aggregate achieved higher compressive strength in comparison to the reference concrete. The reference concrete reached 12.6, 28.2 and 40.6 MPa at 2, 7 and 28 days respectively. The strength after two days of curing of the concrete containing crushed RCA increases as the replacement level increases. Concrete containing 25% of crushed RCA reached a strength of 25.7 MPa, that containing 50% of crushed RCA 30.9 MPa and that containing 75% of crushed RCA 31.3 MPa. A similar relationship applies to concretes containing round RCA. They reached 16.8, 24.7 and 33.8 MPa for 25%, 50% and 75% of the replacement level respectively. After 7 days of curing, for concretes containing crushed RCA, the samples reached a similar compressive strength regardless of the replacement level—43.7, 45.3 and 41.8 MPa for 25%, 50% and 75% respectively. In the case of concretes containing round RCA, the higher the replacement level is, the higher the strength. After 28 days of curing, concretes containing 25% and 50% of crushed RCA reached similar strengths (49.7 and 49.4 MPa). The one containing 75% of crushed RCA reached a lower compressive strength of 44.4 MPa. Concrete containing round RCA reached similar strength after 28 days (48.4–50.0 MPa). As in the case of concretes containing metallurgical sludge waste as a replacement for sand, the increased strength is also due to a decrease of the effective water-cement ratio. Similar results are reported by [82,83,86,87] for both crushed ceramic waste and RCA manufactured from ordinary concrete. Reported strengths are greater or similar to the reference sample.

## 4. Conclusions

To summarize the research, the authors draw attention to the technological possibilities and environmental benefits of using both metallurgical sludge waste in a least processed form and aggregates made with its content.

The first possibility is the disposal of metallurgical sludge waste itself in concrete. Metallurgical sludge waste is originally stored in landfills that take up space that can be used in another, more favorable way. In addition, it can cause soil or groundwater contamination. Therefore, using it as a partial substitute for sand is environmentally beneficial. In addition, the consumption of sand, which is a natural resource, and its reserves are limited and its excavation is regulated in many countries by law. One could go further in this consideration and think that once the lifespan of concrete structures or elements containing metallurgical sludge waste has expired, such concrete could be used as RCA, after crushing.

For the production of ready-mixed concrete and its use on the construction site, there are two possibilities if there is an excess of mix or the mix does not meet the requirements. The first is to add waste and superplasticizer to the mix to improve consistency and then pour it onto a foil-covered surface and after hardening, crush the resulting composite and use it again as crushed RCA. A second option is to use a special admixture to recover the excess concrete mixture and process it into round RCA.

The authors present below the technological aspects of using metallurgical sludge waste and recycled concrete aggregate:

- The use of metallurgical sludge waste or crushed or round RCA to produce concrete deteriorates the consistency. This is associated, in the case of metallurgical sludge waste, with its high water demand, and fineness and, in the case of RCA, with its high absorbability.
- The use of the superplasticizer will result in additional costs, but without its use it will not be possible to use metallurgical sludge waste over 60% or RCA over 50% due to the deteriorated concrete consistency. Additionally, concrete mix recovery agents can increase costs. However, it is certain that the use of waste will have a positive environmental effect.
- The use of metallurgical sludge waste as a replacement for sand in concrete does not affect the air content, density of the concrete mix or the density of the hardened concrete.
- Using RCA as a replacement for coarse aggregate increases the air content in the concrete mix. This is due to the high porosity of RCA. RCA does not have a significant effect on the concrete mix density, but it does affect the density of the hardened concrete. This is due to the fact that RCA has a lower density compared to natural aggregate and, when present in the mix, contains water which evaporates during curing.
- The use of metallurgical sludge waste as a replacement for sand in concrete increases its absorbability and slightly increases the depth of water penetration under pressure. It is connected with its higher porosity. If round RCA is used, the absorbability of the concrete is also increased. The permeability of the concrete increases as well. This may jeopardize the durability of components made of such concrete and requires further testing.
- The use of metallurgical sludge waste as a replacement for sand in concrete improves the mechanical properties after 7 and 28 days of curing up to 60% of metallurgical sludge waste content. However, even replacing 90% of the sand with metallurgical sludge waste does not adversely affect the compressive strength. RCA’s use as a replacement for natural aggregates has a positive effect on the compressive strength of the concrete at all testing dates. The improvement in mechanical properties is associated with a reduction in the effective water-cement ratio. This is due to the high water demand of both metallurgical sludge waste and RCA. Water absorption by the aggregate reduces the amount of water in the cement paste and thus the porosity and strength of the resulting cement matrix.
- According to the advantages and disadvantages the best performance of concrete containing RCA are while crushed RCA is used. However, it has to me mentioned that different methods may be used accordingly to situation.

## Figures and Tables

**Table 1:** Phase composition of Portland cement CEM I 42,5 R [%].

C_3_S	C_2_S	C_3_A	C_4_AF
69.0	9.6	9.4	9.0

**Table 2:** Physical properties of metallurgical sludge waste in comparison to natural sand.

	Bulk Density	Grain Distribution					
		<0.063 mm	0.063–0.125 mm	0.125–0.25 mm	0.25–0.5 mm	0.5–1 mm	1–2 mm
	[g/cm^3^]	[%]					
Metallurgical sludge waste	1.21	64.4	33.5	2.1	0	0	0
Natural sand	1.61	2.1	11.1	30.4	26.5	22.7	7.2

**Table 3:** Chemical composition of metallurgical sludge waste [%].

**Fe_2_O_3_**	**SiO_2_**	**CaO**	**MgO**	**Al_2_O_3_**	**Mn**	**P_2_O_5_**	**Fe(II)**
21.85	6.65	13.56	1.34	1.65	0.60	0.19	20.91
**Na_2_O**	**K_2_O**	**Zn**	**S**	**C**	**Pb**	**Cl^-^**	**Ign. Loss.**
0.17	0.28	2.00	0.33	10.1	0.23	0.06	18.59

**Table 4:** Composition of preliminary RCA for 1 m^3^.

	Recipe 1	Recipe 2	Recipe 3	Recipe 3
Portland cement [kg/m^3^]	450	450	450	450
Water/cement ratio	0.6	0.6	0.7	0.6
Natural aggregate [kg/m^3^]	950	1088	950	950
Metallurgical sludge waste [kg/m^3^]	410	272	410	410
Replacement of sand by dried sludge [%]	30	20	30	30
Superplasticizer [% c.m.]	1.5	1.5	1.5	3.0

**Table 5:** Physical properties of recycled concrete aggregate (RCA) compared to natural aggregate.

	Natural Aggregate	Crushed RCA	Round RCA
Real density [kg/dm^3^]	2.65	2.25	2.20
Absorbability [%]	1.0	4.3	7.6

**Table 6:** Composition of concrete mixes for 1 m^3^ [kg].

Symbol	Cement CEM I 42.5 R	Water	Natural Fine Aggregate	Natural Coarse Aggregate	Metallurgical Sludge Waste	Crushed RCA	Round RCA	Superplasticizer
REF	350	210	448	1342	-	-	-	-
MSW 30	350	210	314	1342	134	-	-	-
MSW 60	350	210	179	1342	269	-	-	-
MSW 60s	350	210	179	1342	269	-	-	7,00
MSW 90	350	210	45	1342	403	-	-	8,75
CR 25	350	210	448	1007	-	335	-	-
CR 50	350	210	448	671	-	671	-	-
CR 75	350	210	448	335	-	1007	-	10,5
RO 25	350	210	448	1007	-	-	335	-
RO 50	350	210	448	671	-	-	671	-
RO 75	350	210	448	335	-	-	1007	10, 5

## References

[1] (2019–2025). Aggregates Market Size, Share Industry Research Report. https://www.grandviewresearch.com/industry-analysis/aggregates-market/methodology.

[2] Peduzzi P. (2014). Sand, Rarer than one Thinks. Environ. Dev..

[3] Sandabbau: Wenn Inseln Und Strände Verschwinden. https://www.spiegel.de/wissenschaft/mensch/sandabbau-wenn-inseln-und-straende-verschwinden-a-1221226.html.

[4] Global Aggregates Market to be Worth US$608.9bn by 2026. https://www.aggregateresearch.com/news/global-aggregates-market-to-be-worth-us608-9bn-by-2026/.

[5] Langer W., Drew L., Sachs J. (2004). Aggregate and the Environment.

[6] Delannoy A. (2019). The Contribution of Recycled and Secondary Materials to Total Aggregates Supply in Great Britain.

[7] Evangelista L., Guedes M., De Brito J., Ferro A.C., Pereira M.F. (2015). Physical, chemical and mineralogical properties of fine recycled aggregates made from concrete waste. Constr. Build. Mater..

[8] Chauhan S.S. (2010). Mining, Development and Environment: A Case Study of Bijolia Mining Area in Rajasthan, India. J. Hum. Ecol..

[9] Smith J. (2018). Sand storm. New Sci..

[10] Saviour M.N. (2012). Environmental Impact of Soil and Sand Mining: A reviev. Int. J. Sci. Environ. Technol..

[11] Inside the Deadly World of INDIA’S Sand Mining Mafia. https://www.nationalgeographic.com/environment/2019/06/inside-india-sand-mining-mafia/.

[12] Beiser V. (2018). The World in a Grain: The Story of Sand and How It Transformed Civilization.

[13] Neumann F., Curbach M. (2018). Thermal treatment of desert sand to produce construction material. MATEC Web Conf..

[14] Zhang M., Liu H., Sun S., Chen X., Doh S.I. (2019). Dynamic Mechanical Behaviors of Desert Sand Concrete (DSC) after Different Temperatures. Appl. Sci..

[15] Kumar Buraka A. (2016). Mechanical Properties of Concrete with Marine Sand as Partial Replacement of Fine Aggregate. J. Eng. Res. Appl..

[16] Limeira J., Agullo L., Etxeberria M. (2010). Dredged marine sand in concrete: An experimental section of a harbor pavement. Constr. Build. Mater..

[17] Paldyna J., Krasnodebska-Ostrega B., Kregielewska K., Kowalska J., Jedynak L., Golimowski J., Grobelski T., Farbiszewska-Kiczma J., Farbiszewska T. (2013). The assessment of environmental pollution caused by mining and metallurgy wastes from highly polluted post-industrial regions in Southern Poland. Environ. Earth Sci..

[18] Dhal B., Thatoi H.N., Das N.N., Pandey B.D. (2013). Chemical and microbial remediation of hexavalent chromium from contaminated soil and mining/metallurgical solid waste: A review. J. Hazard. Mater..

[19] Vaněk A., Grösslová Z., Mihaljevič M., Ettler V., Trubač J., Chrastný V., Penížek V., Teper L., Cabala J., Voegelin A. (2018). Thallium isotopes in metallurgical wastes/contaminated soils: A novel tool to trace metal source and behavior. J. Hazard. Mater..

[20] Moutsatsou A., Gregou M., Matsas D., Protonotarios V. (2006). Washing as a remediation technology applicable in soils heavily polluted by mining-metallurgical activities. Chemosphere.

[21] Katzer J. (2013). Strength performance comparison of mortars made with waste fine aggregate and ceramic fume. Constr. Build. Mater..

[22] Hornakova M., Katzer J., Kobaka J., Konecny P. (2019). Lightweight SFRC Benefitting from a Pre-Soaking and Internal Curing Process. Materials.

[23] Malešev M., Radonjanin V., Marinković S. (2010). Recycled Concrete as Aggregate for Structural Concrete Production. Sustainability.

[24] Pawluczuk E., Kalinowska-Wichrowska K., Bołtryk M., Jiménez J., Fernández J. (2019). The Influence of Heat and Mechanical Treatment of Concrete Rubble on the Properties of Recycled Aggregate Concrete. Materials.

[25] Yang S. (2018). Effect of Different Types of Recycled Concrete Aggregates on Equivalent Concrete Strength and Drying Shrinkage Properties. Appl. Sci..

[26] Sadowska-Buraczewska B., Barnat-Hunek D., Szafraniec M. (2020). Influence of Recycled High-Performance Aggregate on Deformation and Load-Carrying Capacity of Reinforced Concrete Beams. Materials.

[27] McNeil K., Kang T.H.K. (2013). Recycled Concrete Aggregates: A Review. Int. J. Concr. Struct. Mater..

[28] Fan C.-C., Huang R., Hwang H., Chao S.-J. (2015). The Effects of Different Fine Recycled Concrete Aggregates on the Properties of Mortar. Materials.

[29] Pavlů T., Kočí V., Hájek P. (2019). Environmental Assessment of Two Use Cycles of Recycled Aggregate Concrete. Sustainability.

[30] Adessina A., Ben Fraj A., Barthélémy J.F., Chateau C., Garnier D. (2019). Experimental and micromechanical investigation on the mechanical and durability properties of recycled aggregates concrete. Cem. Concr. Res..

[31] Wang H., Sun X., Wang J., Monteiro P. (2016). Permeability of Concrete with Recycled Concrete Aggregate and Pozzolanic Materials under Stress. Materials.

[32] Cartuxo F., de Brito J., Evangelista L., Jiménez J., Ledesma E. (2016). Increased Durability of Concrete Made with Fine Recycled Concrete Aggregates Using Superplasticizers. Materials.

[33] Arredondo-Rea S., Corral-Higuera R., Gómez-Soberón J., Gámez-García D., Bernal-Camacho J., Rosas-Casarez C., Ungsson-Nieblas M. (2019). Durability Parameters of Reinforced Recycled Aggregate Concrete: Case Study. Appl. Sci..

[34] Bao J., Li S., Zhang P., Ding X., Xue S., Cui Y., Zhao T. (2020). Influence of the incorporation of recycled coarse aggregate on water absorption and chloride penetration into concrete. Constr. Build. Mater..

[35] Sas W., Głuchowski A., Gabryś K., Soból E., Szymański A. (2016). Deformation Behavior of Recycled Concrete Aggregate during Cyclic and Dynamic Loading Laboratory Tests. Materials.

[36] Głuchowski A., Sas W., Dzięcioł J., Soból E., Szymański A. (2018). Permeability and Leaching Properties of Recycled Concrete Aggregate as an Emerging Material in Civil Engineering. Appl. Sci..

[37] Tam V.W.Y., Soomro M., Evangelista A.C.J. (2018). A review of recycled aggregate in concrete applications (2000–2017). Constr. Build. Mater..

[38] Wang X., Chin C., Xia J. (2019). Material Characterization for Sustainable Concrete Paving Blocks. Appl. Sci..

[39] Galan J.J., Silva L.M., Pérez I., Pasandín A.R. (2019). Mechanical Behavior of Hot-Mix Asphalt Made with Recycled Concrete Aggregates from Construction and Demolition Waste: A Design of Experiments Approach. Sustainability.

[40] Acosta Álvarez D., Alonso Aenlle A., Tenza-Abril A.J., Ivorra S. (2019). Influence of Partial Coarse Fraction Substitution of Natural Aggregate by Recycled Concrete Aggregate in Hot Asphalt Mixtures. Sustainability.

[41] Martinho F., Picado-Santos L., Capitão S. (2018). Feasibility Assessment of the Use of Recycled Aggregates for Asphalt Mixtures. Sustainability.

[42] Hou Y., Ji X., Li J., Li X. (2018). Adhesion between Asphalt and Recycled Concrete Aggregate and Its Impact on the Properties of Asphalt Mixture. Materials.

[43] Kox S., Vanroelen G., Van Herck J., de Krem H., Vandoren B. (2019). Experimental evaluation of the high-grade properties of recycled concrete aggregates and their application in concrete road pavement construction. Case Stud. Constr. Mater..

[44] Tang W., Khavarian M., Yousefi A., Chan R.W.K., Cui H. (2019). Influence of Surface Treatment of Recycled Aggregates on Mechanical Properties and Bond Strength of Self-Compacting Concrete. Sustainability.

[45] Martínez-García R., Guerra-Romero M.I., Pozo J.M.M., Brito J., de Juan-Valdés A. (2020). Recycling Aggregates for Self-Compacting Concrete Production: A Feasible Option. Materials.

[46] Sasanipour H., Aslani F. (2020). Durability properties evaluation of self-compacting concrete prepared with waste fine and coarse recycled concrete aggregates. Constr. Build. Mater..

[47] Mesgari S., Akbarnezhad A., Xiao J.Z. (2020). Recycled geopolymer aggregates as coarse aggregates for Portland cement concrete and geopolymer concrete: Effects on mechanical properties. Constr. Build. Mater..

[48] Nobre J., Bravo M., de Brito J., Duarte G. (2020). Durability performance of dry-mix shotcrete produced with coarse recycled concrete aggregates. J. Build. Eng..

[49] Duarte G., Bravo M., de Brito J., Nobre J. (2019). Mechanical performance of shotcrete produced with recycled coarse aggregates from concrete. Constr. Build. Mater..

[50] Gales J., Parker T., Cree D., Green M. (2016). Fire Performance of Sustainable Recycled Concrete Aggregates: Mechanical Properties at Elevated Temperatures and Current Research Needs. Fire Technol..

[51] Kianimehr M., Shourijeh P.T., Binesh S.M., Mohammadinia A., Arulrajah A. (2019). Utilization of recycled concrete aggregates for light-stabilization of clay soils. Constr. Build. Mater..

[52] Lu J.X., Yan X., He P., Poon C.S. (2019). Sustainable design of pervious concrete using waste glass and recycled concrete aggregate. J. Clean. Prod..

[53] Salahuddin H., Qureshi L.A., Nawaz A., Raza S.S. (2020). Effect of recycled fine aggregates on performance of Reactive Powder Concrete. Constr. Build. Mater..

[54] Cabrera M., Galvín A.P., Agrela F. (2019). Leaching issues in recycled aggregate concrete. New Trends in Eco-Efficient and Recycled Concrete.

[55] Neville A.M. (1995). Properties of Concrete.

[56] Kong D., Chen M., Xie J., Zhao M., Yang C. (2019). Geometric Characteristics of BOF Slag Coarse Aggregate and its Influence on Asphalt Concrete. Materials.

[57] Lee K.M., Lee H.K., Lee S.H., Kim G.Y. (2006). Autogenous shrinkage of concrete containing granulated blast-furnace slag. Cem. Concr. Res..

[58] Al-Jabri K.S., Hisada M., Al-Oraimi S.K., Al-Saidy A.H. (2009). Copper slag as sand replacement for high performance concrete. Cem. Concr. Compos..

[59] Prem P.R., Verma M., Ambily P.S. (2018). Sustainable cleaner production of concrete with high volume copper slag. J. Clean. Prod..

[60] Lam M., Jaritngam S., Le D.-H. (2018). EAF Slag Aggregate in Roller-Compacted Concrete Pavement: Effects of Delay in Compaction. Sustainability.

[61] Sosa I., Thomas C., Polanco J.A., Setién J., Tamayo P. (2020). High Performance Self-Compacting Concrete with Electric Arc Furnace Slag Aggregate and Cupola Slag Powder. Appl. Sci..

[62] Adediran A., Yliniemi J., Illikainen M. (2019). Fayalite Slag as Binder and Aggregate in Alkali-Activated Materials—Interfacial Transition Zone Study. Multidiscip. Digit. Publ. Inst. Proc..

[63] Demirboǧa R., Gül R. (2006). Production of high strength concrete by use of industrial by-products. Build. Environ..

[64] Thakur I.C., Kumar S., Singh J.P. (2016). Assessment of the Properties of Cement and Mortar using GGBS. Int. J. Innov. Res. Sci. Eng. Technol..

[65] Patra R.K., Mukharjee B.B. (2017). Influence of incorporation of granulated blast furnace slag as replacement of fine aggregate on properties of concrete. J. Clean. Prod..

[66] Rashad A.M., Sadek D.M., Hassan H.A. (2016). An investigation on blast-furnace stag as fine aggregate in alkali-activated slag mortars subjected to elevated temperatures. J. Clean. Prod..

[67] Netinger I., Bjegović D., Vrhovac G. (2011). Utilisation of steel slag as an aggregate in concrete. Mater. Struct..

[68] Yellishetty M., Karpe V., Reddy E.H., Subhash K.N., Ranjith P.G. (2008). Reuse of iron ore mineral wastes in civil engineering constructions: A case study. Resour. Conserv. Recycl..

[69] Ismail Z.Z., AL-Hashmi E.A. (2008). Reuse of waste iron as a partial replacement of sand in concrete. Waste Manag..

[70] Alwaeli M. (2016). The implementation of scale and steel chips waste as a replacement for raw sand in concrete manufacturing. J. Clean. Prod..

[71] Wiesława N.W., Barbara T., Sylwia D. (2015). The properties of cement pastes and mortars processed with some heavy metal nitrates containing solutions. Proceedings of the Procedia Engineering.

[72] Wei Y.-L., Ko G.-W. (2017). Recycling steel wastewater sludges as raw materials for preparing lightweight aggregates. J. Clean. Prod..

[73] Gawlicki M., Czamarska D. (1992). Effect of ZnO on the hydration of Portland cement. J. Therm. Anal..

[74] Trezza M.A. (2007). Hydration study of ordinary portland cement in the presence of zinc ions. Mater. Res..

[75] Ghannam S., Najm H., Vasconez R. (2016). Experimental study of concrete made with granite and iron powders as partial replacement of sand. Sustain. Mater. Technol..

[76] Achal J., Nitin T. (2018). Effect of partial replacement of sand by glass powder and steel powder over the properties of concrete: Implementation. Int. J. Adv. Res..

[77] Natarajan S., Murugesan P. (2019). Synergistic effect of marble powder and green sand on the mechanical properties of metakaolin-cement concrete. Materials.

[78] Kabeer K.I.S.A., Vyas A.K. (2018). Utilization of marble powder as fine aggregate in mortar mixes. Constr. Build. Mater..

[79] Gallias J.-L., Bigas J.-P. (2002). Effect of fine mineral additions on granular packing of cement mixtures Valorization of Local Mineral Admixtures in Concretes View project Non destructive testing of reinforced concrete structures View project Effect of fine mineral additions on granular. Mag. Concr. Res..

[80] Nagar B., Bhargava V.P. (2016). Experimental study on effects of sludge waste in concrete. Int. J. Eng. Sci. Res. Technol..

[81] Kubissa J., Koper M., Koper W., Kubissa W., Koper A. (2015). Water demand of concrete recycled aggregates. Proceedings of the Procedia Engineering.

[82] Butler L., West J.S., Tighe S.L. (2011). The effect of recycled concrete aggregate properties on the bond strength between RCA concrete and steel reinforcement. Cem. Concr. Res..

[83] Safiuddin M., Alengaram U.J., Salam M.A., Jumaat M.Z., Jaafar F.F., Saad H.B. (2011). Properties of high-workability concrete with recycled concrete aggregate. Mater. Res..

[84] Khalid F.S., Azmi N.B., Sumandi K.A.S.M., Mazenan P.N. (2017). Mechanical properties of concrete containing recycled concrete aggregate (RCA) and ceramic waste as coarse aggregate replacement. The AIP Conference Proceedings.

[85] Shayan A., Xu A. (2003). Performance and Properties of Structural Concrete made with Recycled Concrete Aggregate. ACI Mater. J..

[86] Qasrawi H., Shalabi F., Asi I. (2009). Use of low CaO unprocessed steel slag in concrete as fine aggregate. Constr. Build. Mater..

[87] Neno C., Brito J.D., Veiga R. (2014). Using fine recycled concrete aggregate for mortar production. Mater. Res..

